# Temporal and Spatial Gene Expression Dynamics in Neonatal HI Hippocampus with Focus on Arginase

**DOI:** 10.3390/cells15030253

**Published:** 2026-01-28

**Authors:** Michael A. Smith, Eesha Natarajan, Carlos Lizama-Valenzuela, Thomas Arnold, David Stroud, Amara Larpthaveesarp, Cristina Alvira, Jeffrey R. Fineman, Donna M. Ferriero, Emin Maltepe, Fernando Gonzalez, Jana K. Mike

**Affiliations:** 1 Department of Pediatrics, University of California San Francisco, San Francisco, CA 94143, USA; michael.smith2@ucsf.edu (M.A.S.); carlos.lizamavalenzuela@ucsf.edu (C.L.-V.); thomas.arnold@ucsf.edu (T.A.); david.stroud@ucsf.edu (D.S.); amara.larpthaveesarp@ucsf.edu (A.L.); cristina.alvira@ucsf.edu (C.A.); jeff.fineman@ucsf.edu (J.R.F.); donna.ferriero@ucsf.edu (D.M.F.); emin.maltepe@ucsf.edu (E.M.); fernando.gonzalez@ucsf.edu (F.G.); jana.mike@ucsf.edu (J.K.M.); 2 Initiative for Pediatric Drug and Device Development, San Francisco, CA 94158, USA; 3 Department of Neurology, Weill Institute for Neurosciences, University of California San Francisco, San Francisco, CA 94158, USA; 4 Department of Biomedical Sciences, University of California San Francisco, San Francisco, CA 94143, USA

**Keywords:** neonatal, hypoxia, brain, transcriptomics, arginase

## Abstract

**Highlights:**

**What are the main findings?**

- This study defines the temporal cellular architecture of the neonatal hippocampal response to hypoxic–ischemic injury by integrating cell-type-resolved transcriptomics and spatial mapping at Day 1 and Day 5.
- Early microglia display an efferocytic and inflammatory-resolving program, whereas subacute microglia shift toward extracellular matrix remodeling and fibrotic pathways.

**What are the implications of the main findings?**

- These findings identify distinct phases of microglial activation and highlight therapeutic windows for modulating reparative microglial states.

**Abstract:**

Background: Hypoxic–ischemic (HI) brain injury triggers a dynamic, multi-phase response involving early microglial efferocytosis followed by extracellular matrix (ECM) deposition and scar formation. Arginase-1 (ARG1), a key enzyme in tissue repair, is implicated in both processes, yet its role in neonatal microglia remains poorly defined. We characterize ARG1-linked pathways in neonatal microglia, identifying distinct efferocytic and fibrotic phases post-HI. Methods: HI was induced in P9 mice using the Vannucci model, and brains were collected at 24 h (D1) and 5 days (D5). Spatially resolved single-cell transcriptomics (seqFISH) was performed using a targeted panel enriched for microglial, ARG1-pathway, efferocytosis, and profibrotic genes. Cell segmentation, clustering, and spatial mapping were conducted using Navigator and Seurat. Differential expression, GSEA, and enrichment analyses were used to identify time- and injury-dependent pathways. Results: Spatial transcriptomics identified 12 transcriptionally distinct cell populations with preserved neuroanatomical organization. HI caused the expansion of microglia and astrocytes and the loss of glutamatergic neurons by D5. Microglia rapidly activated regenerative and profibrotic programs—including *TGF-β*, *PI3K–Akt*, cytoskeletal remodeling, and migration—driven by early DEGs such as *Cd44*, *Reln*, *TGF-β1*, and *Col1a2*. By D5, microglia adopted a collagen-rich fibrotic state with an upregulation of *Bgn*, *Col11a1*, *Anxa5*, and *Npy*. Conclusion: Neonatal microglia transition from early efferocytic responses to later fibrotic remodeling after HI, driven by the persistent activation of *PI3K–Akt*, *TGF-β*, and *Wnt/FZD4* pathways. These findings identify microglia as central regulators of neonatal scar formation and highlight therapeutic targets within ARG1-linked signaling.

## 1. Introduction

Neonatal hypoxic–ischemic (HI) brain injury remains a major clinical challenge, with an urgent need for new therapeutic strategies. Advancing effective treatments requires a deeper understanding of the complex and dynamic biological responses to HI in the developing brain. Neonatal brain HI is characterized by a multi-phase pathological process with early efferocytosis and later extracellular matrix (ECM)-driven scarring [1,2,3]. Arginase-1 (ARG1), an enzyme with established roles in microglial tissue repair, has emerged as a key participant in both efferocytosis and scar formation [4]. Elevated ARG1 levels have been associated with improved neurological outcomes [5], and, in the adult brain, ARG1 contributes to microglial polarization [6], regulation of oxidative stress [7], apoptosis [8], efferocytosis [9], ECM remodeling [10], scar formation [11], and neurite growth [12]. However, despite these established functions in the mature brain, the developmental roles of ARG1 signaling in microglia remain poorly defined.

Recent evidence shows that ARG1 is robustly expressed during development in a subpopulation of microglia with regional enrichment in the ventral brain [13,14]. Our previous work demonstrated that, although ARG1 expression normally decreases as microglia mature, HI reactivates ARG1 in a select microglial population at 12 h at the injury core [14]. The hippocampus, particularly the CA1 and CA3 subfields, is uniquely vulnerable to the metabolic failure induced by HI, resulting in delayed yet substantial neuronal loss [15]. Injury to this region during the neonatal period is strongly associated with the permanent cognitive impairments, learning disabilities, and memory deficits frequently seen in children who survive HI [16]. ARG1 may contribute to HI pathology through two primary pathways. One involves the *IL-4–STAT6–PPARγ–ARG1* axis, in which ARG1 microglia support efferocytosis by engulfing dying cells and removing debris, an essential step for resolving inflammation and initiating tissue repair [17,18,19]. A second involves the *TGF-β–ARG1–COL1A2* pathway, where TGF-β is the master regulator of fibrosis and a strong inducer of ARG1 [20,21,22,23,24]. ARG1 generates proline precursors required for collagen production [20,22] and enhances COL1A2 production, collectively driving ECM accumulation and fibroblast activation in tissue fibrogenesis [25].

Through polyamine synthesis, ARG1 contributes to the recruitment and migration of various cell types to the injury site, such as vascular smooth muscle cells and fibroblasts [26,27], facilitating scar formation. The CNS tissue scar is a compartmentalized, multicellular structure that ideally seals off the inflammation locally and forms a supportive environment for tissue remodeling. Although this scarring stabilizes damaged tissue, it can also impede regeneration [28].

Based on this accumulating evidence for ARG1 critically shaping neonatal HI-induced neuroinflammation and repair, we hypothesized that ARG1 is a key regulator of microglial response to neonatal HI. We employed spatially resolved single-cell transcriptomics (seqFISH, a fluorescence in situ hybridization) focused on microglia in the hippocampus to characterize these ARG1-associated injury responses.

## 2. Methods

### 2.1. Animals

All animal research was approved by the University of California San Francisco Institutional Animal Care and Use Committee and was performed in accordance with the NIH guidelines for the Care and Use of Laboratory Animals. We used the wild-type C57BL/6 mice strains (Ferriero Laboratory, San Francisco, CA, USA). Our study compared samples from HI and SHAM groups (n = 3 per group) across two timepoints, day 1 post HI (D1, corresponding to postnatal day 10, P10) and day 5 post HI (D5, corresponding to postnatal day 15, P15).

Spatial transcriptomics remains technically challenging and high-cost, which has led most published studies to use a limited number of biological replicates (typically n ≈ 3–4 per condition) while maximizing within-sample coverage and integration with complementary modalities. Following this established standard, we prioritized data quality per animal rather than increasing n at the expense of spatial resolution or transcript detection. Each replicate yielded thousands of high-quality cells/spots, and unsupervised analyses demonstrated strong concordance across biological replicates within each group, including clear separation by condition and time point with minimal batch effects. This level of reproducibility is consistent with prior microglial and brain injury transcriptomic studies using n = 3 per group. Although a larger cohort could further increase the power to detect subtle transcriptional differences, the replicated gene-level changes, convergent pathway enrichment results, and orthogonal validation collectively support the robustness of the major findings reported here.

### 2.2. Induction of Hypoxia–Ischemia

Neonatal HI was induced at P9 using the Vannucci procedure, which is a well-established model to study HI in the neonatal brain. Briefly, the left common carotid artery (CCA) was occluded by coagulation under isoflurane anesthesia (4% isoflurane, balance O2). The mice were recovered for 1 h with their dam. The unilateral HI insult was induced by exposure to 50 min of hypoxia in a humidified chamber at 37 °C with 10% O2/balance N2. The mice were returned to the dam and euthanized at D1 (expecting the most robust efferocytosis), and D5 (early scar with clear glial borders) after the procedure. Sham mice were exposed to anesthesia and incision but no CCA coagulation or hypoxia were used as controls.

### 2.3. Tissue Preparation

#### Spatial Genomics Gene Positioning System (GenePS)

We focused spatial genomics analysis on the damaged hemisphere only because it experiences both ischemia and hypoxia, which are the conditions relevant to our study. The contralateral hemisphere experiences hypoxia alone in the Vannucci model, which can produce significant molecular and genetic changes, making it potentially difficult to compare and interpret with our experimental condition of HI. Mouse brains were embedded in OCT and snap-frozen. A 20 µm coronal section from bregma −1.0 to −2.5 mm was sectioned onto specialized coverslips (Spatial Genomics, Pasadena, CA, USA, Catalog No. 10200003) and fixed for 15 min at room temperature with 4% paraformaldehyde (PFA) in 1× PBS. Tissue processing followed the Spatial Genomics Frozen Tissue Sample Preparation Guide, and sections were hybridized with the Spatial Genomics Mouse Brain Mapper Panel, supplemented with additional genes related to the *ARG1* pathway: *ARG* isoforms (*ARG1* and *ARG2*), microglial markers (*TMEM119* and *P2RY12*), and genes involved in both the regulation of the *ARG* pathway and brain efferocytosis (*Rac1*, *STAT6*, *PPARγ*) or fibrosis (*Col1a2*, *TGF-β1*, *TGF-β2*, *IFNGR1*, *IFNGR2*) were selected.

For imaging and decoding, tissue sections were processed and then loaded on the GenePS instrument (GenePS v1; Spatial Genomics, Inc.), an automated platform for image acquisition, reagent delivery, and data processing. The GenePS was loaded with samples, decode plates, and buffers following the Instrument User Guide, and regions of interest that captured the full tissue section were selected based on DAPI-stained nuclei. The automated workflow then captured high-magnification single z-plane images across samples, followed by iterative rounds of decode probe hybridization, imaging, and signal removal until all hybridization cycles were complete. Raw image files were aligned across rounds and processed on the GenePS instrument to localize RNA signals. Subsequent analysis in Spatial Genomics’ Navigator software v0.8.0a5 enabled transcript decoding and cell segmentation, using a machine learning-based algorithm to delineate nuclei and then cell boundaries were expanded beyond the detected DAPI staining. Transcripts were decoded and assigned to cells, generating cell-by-gene matrices for each sample with an average of 74,504 cells analyzed per sample (Table S1). The final outputs—including expression matrices, cell coordinates, transcript lists, DAPI images, and segmentation masks—provided high-resolution spatial and transcriptional data for downstream analysis.

### 2.4. Data Analysis

Data analysis was performed using Matlab (The Mathworks, Natick, MA, USA, R2024a). To ensure data quality, we applied cutoffs for each sample based on total transcript counts per cell, excluding the top 1 percentile and bottom 3 percentiles (Figure S1A). Seurat v5.1.0 was used to merge [29], visualize, and cluster the samples. Cell area-normalized counts were used to generate uniform manifold approximation and projection plots (UMAPs) of the combined dataset and confirmed the absence of significant batch effects between experimental conditions (day or injury, Figure S1B) or samples (Figure S1C). We performed pseudobulk differentially expressed gene analysis (DEG) on all cells for SHAM vs. HI using Muscat v1.18 [30]. The optimal clustering resolution parameter for Louvain clustering was determined using random forests and a silhouette score-based assessment of clustering validity and subject-wise cross-validation [31]. This procedure is described in greater detail by George et al. [32]. Clustering at the reproducible resolution parameter of 0.1 and 25 principal components resulted in 12 clusters. Using a Wilcoxon rank sum test we identified the top genes for each cluster, using them to query CellKB and cell types. Pseudobulk DEG analysis was performed for all clusters for the whole slides and separately for the left hippocampus region, and genes with Benjamini–Hochberg-adjusted *p*-value < 0.05 were considered significantly differentially expressed. Gene set enrichment analysis (GSEA) was performed in fgsea v1.30.0 and over-representation analysis (ORA) implemented in clusterProfiler v4.12.6, executed in R v4.4.1. Pathway-level enrichment was assessed using GSEA and ORA across GO, WikiPathways, and PFOCR gene sets. For enrichment analyses, significance was defined as FDR q-value < 0.25, even when the corresponding nominal or adjusted *p*-values exceeded 0.05, consistent with standard GSEA practice for discovery-oriented pathway interpretation. In parallel, interactive enrichment analysis was performed, integrating DEG statistics (log_2_ fold change, adjusted *p*-value < 0.05, rank) with curated pathway databases to generate a complementary set of enriched pathways and to quantify individual driver gene contributions. This allowed the interrogation of pathway activity at multiple resolution levels, capturing both global enrichment trends and the specific genes driving these processes.

## 3. Results

### 3.1. Spatial Transcriptomics Reveals a Global Architecture with Region-Specific Responses Post-HI

**Figure 1:**
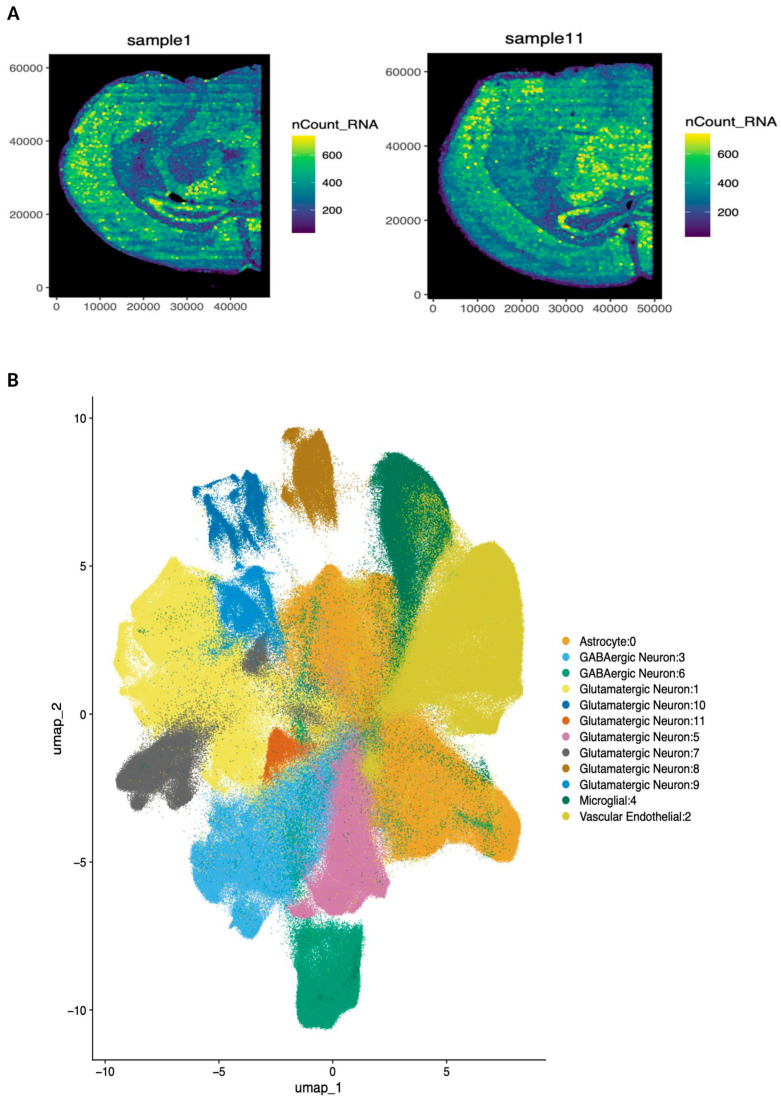

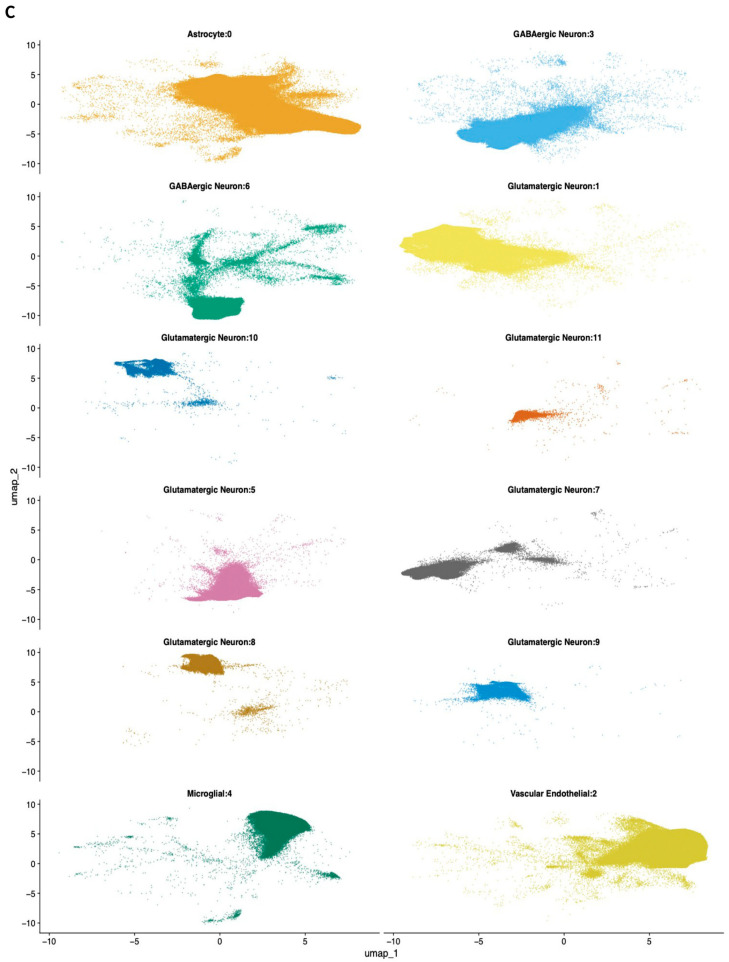

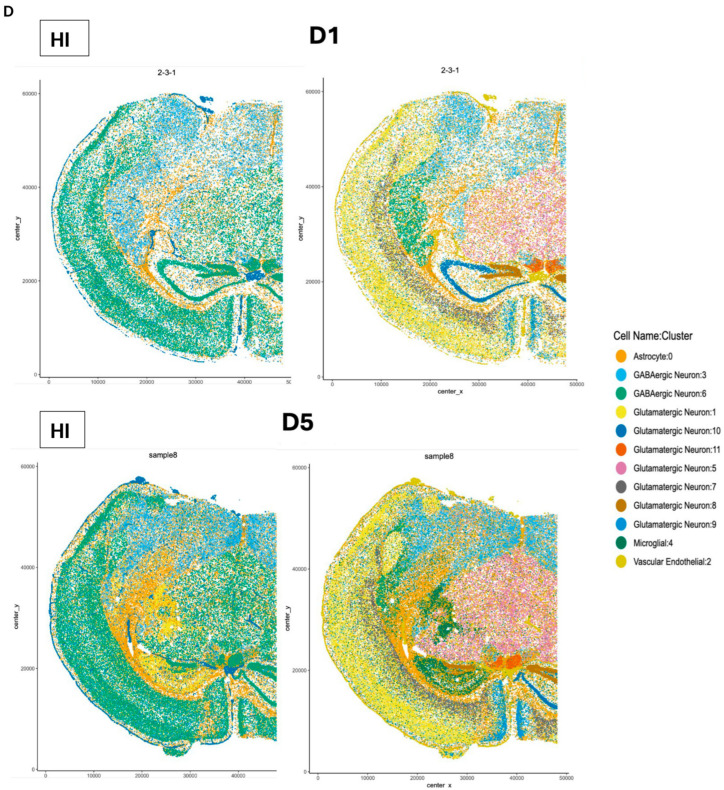

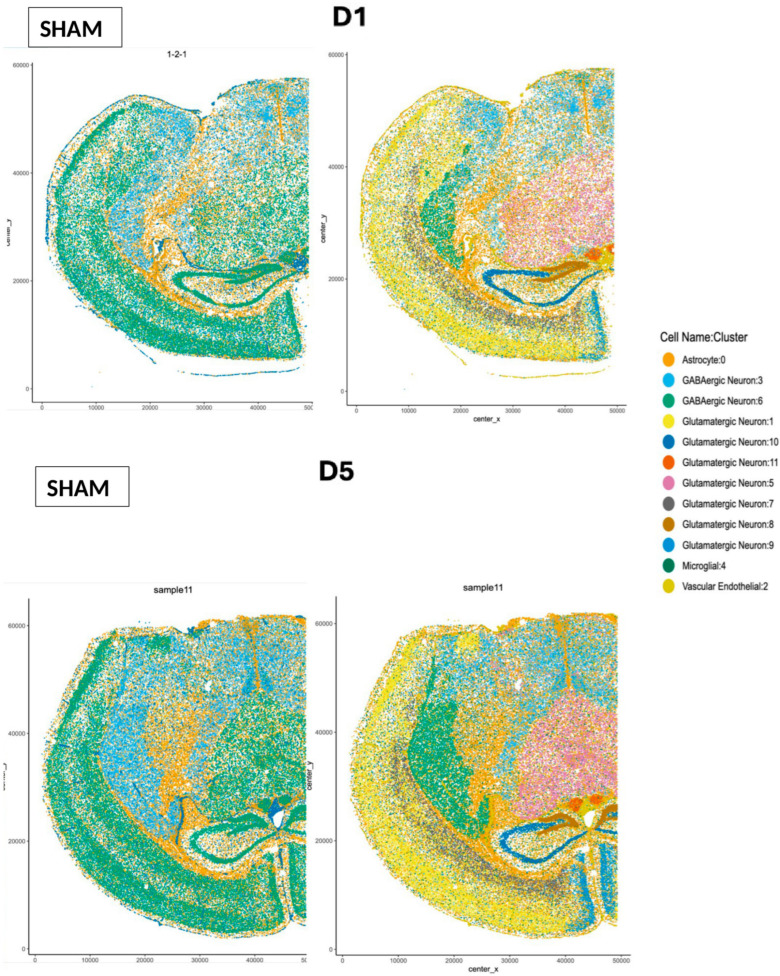
Spatial cell-type organization in HI and sham mouse brains at D1 and D5. (**A**) Spatial distribution of RNA counts. (**B**) UMAP with transcriptionally defined cell clusters. (**C**) Clusters of cellular subtypes, including astrocytes, microglia, vascular endothelial cells, GABAergic neurons, and multiple glutamatergic neurons. (**D**) Spatial projection of annotated cell types onto tissue sections from HI and sham brains at D1 and D5, demonstrating preserved neuroanatomical structure and regionally distinct cell distributions.

High-resolution spatial transcriptomics provides a powerful tool through which we can define cellular diversity while preserving the anatomical architecture of the brain. In our study, the seqFISH technology enabled spatial assessment of RNA counts of n = 3 animals each from the HI and sham groups at D1 and D5 (Figure 1A). Using a curated panel of well-validated marker genes, we assigned cell type identities across the dataset. Unsupervised clustering revealed 12 transcriptionally distinct populations (Figure 1B). For each cluster, we identified defining marker genes and leveraged CellKB for orthogonal validation, resulting in the annotation of 12 discrete cell types (Figure 1C). Joint clustering of all samples revealed transcriptionally distinct populations corresponding to astrocytes, microglia, vascular endothelial cells, GABAergic neurons, and multiple glutamatergic neuronal subtypes (Figure 1C). Spatial projection of these clusters onto tissue sections demonstrated fundamental neuroanatomical organization, with neurons localized to expected cortical and hippocampal areas, astrocytes broadly distributed throughout the parenchyma, and endothelial cells outlining vascular structures and meninges (Figure 1D). The multiple clusters of glutamatergic neurons corresponded to distinct spatial regions even though no spatial information was used to generate them (Figure 1D).

### 3.2. Cell-Type-Specific Population Dynamics in Hippocampus Post-HI

**Figure 2:**
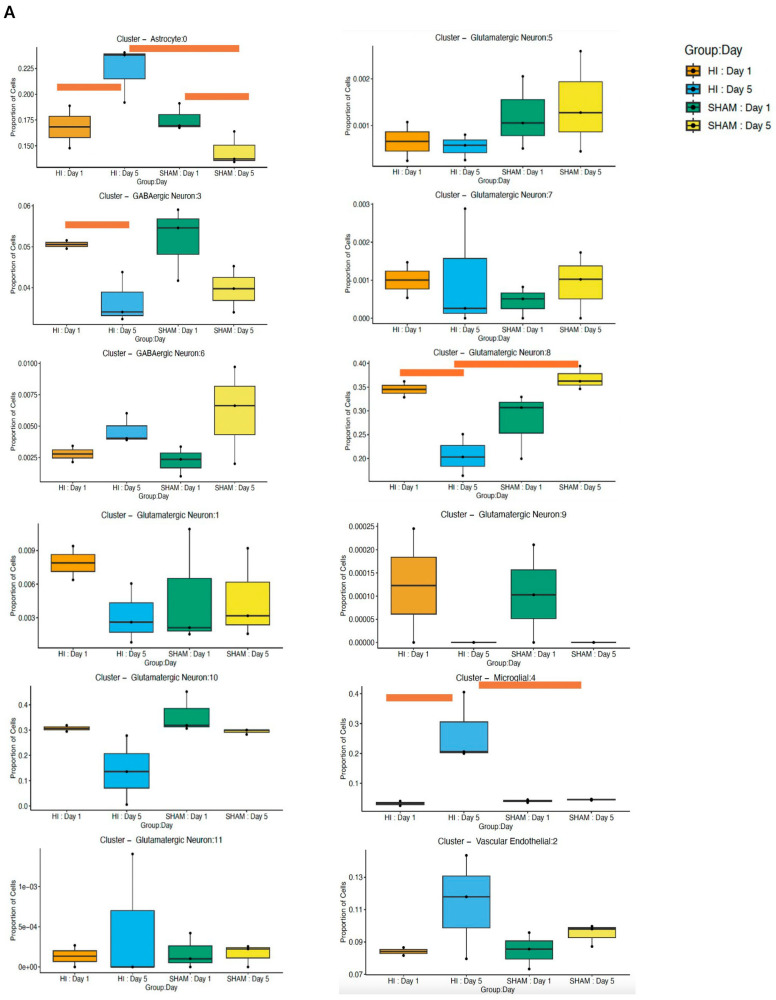

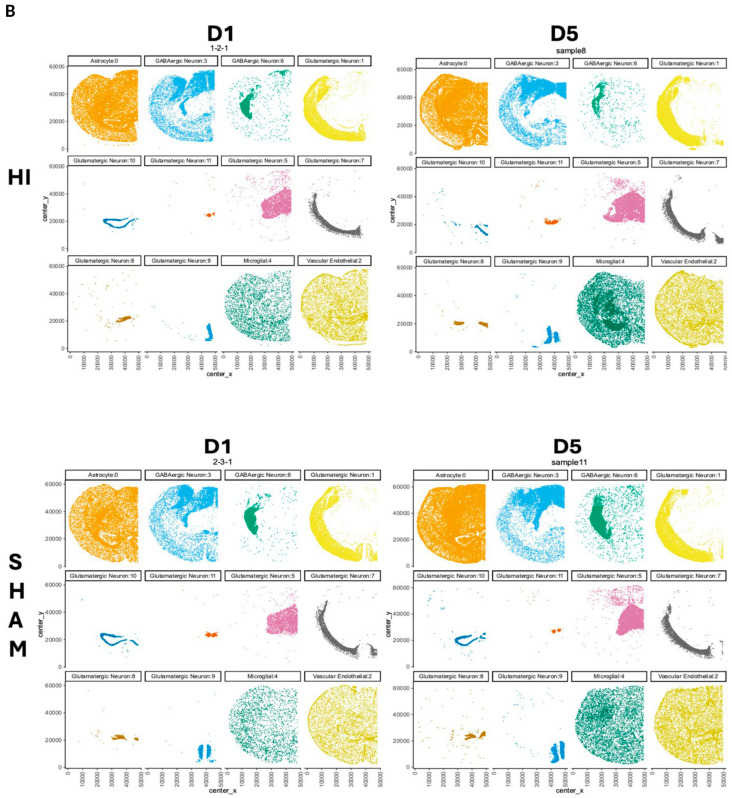
Cell-type composition changes in hippocampal tissue post-HI at D1 and D5: (**A**) Proportion of cells per annotated cluster across experimental conditions (HI D1, HI D5, sham D1, sham D5). Boxplots display changes in relative abundance for major hippocampal cell populations, including astrocytes, GABAergic neurons, multiple glutamatergic neuronal subclusters, microglia, and vascular endothelial cells. Colored bars indicate significant differences across groups (*p* < 0.05). (**B**) Spatial distribution maps of cell clusters in hippocampal sections from HI (left panels) and SHAM (right panels) animals at D1 and D5. Each cell type is visualized by its spatial coordinates and colored according to its assigned cluster label.

Cellular proportion analysis in the hippocampus (Figure 2A) revealed shifts in specific cell populations across development and in response to HI. By D5, HI resulted in a significant expansion of the astrocyte population relative to the early post-injury time point (*p* = 0.007) and to age-matched sham controls (*p* = 3.8 × 10^−7^). Microglia also exhibited a pronounced peak at D5 post-HI, surpassing both HI D1 (*p* = 1.2 × 10^−18^) and sham D5 levels (*p* = 1.98 × 10^−17^). In contrast, glutamatergic neurons cluster 8 declined in abundance by D5 post-HI when compared with HI D1 (*p* = 7.46 × 10^−4^) and sham D5 (*p* = 5.5 × 10^−6^), indicating a selective loss of neurons after injury. Cell-type-specific spatial maps further demonstrated regionally concentrated differences between conditions. In HI samples, microglia and astrocytes showed focal enrichment within vulnerable hippocampal and striatal injury zones at D5 (Figure 2B).

### 3.3. Developmental Transcriptomic Changes in Microglia in P10 and P15 Sham Mice

**Figure 3:**
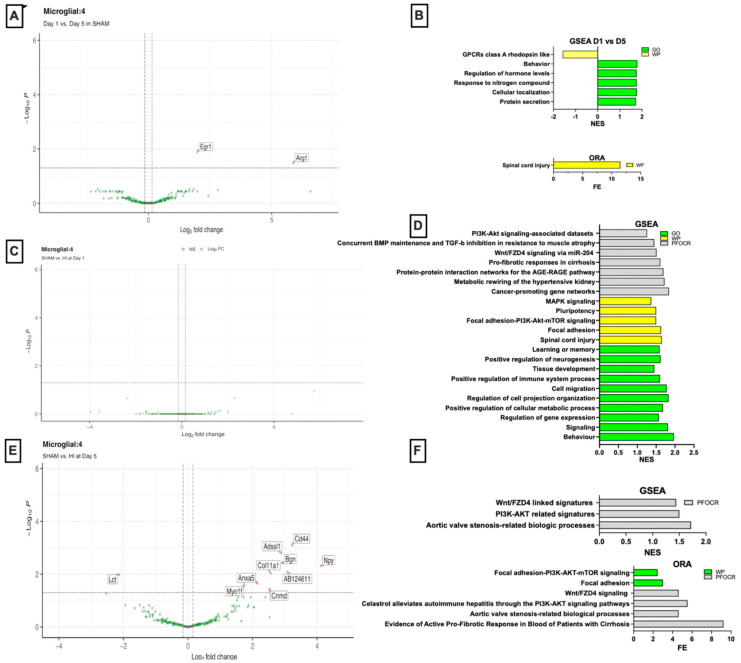
Transcriptomic shifts in hippocampal microglia. (**A**) Volcano plot of DEGs in microglia assessing developmental changes at D1 vs. D5. EGR1 and ARG1 show significant temporal modulation during normal maturation (*p* adj < 0.05). (**B**) GSEA and ORA comparing D1 vs. D5 in sham microglia demonstrates enrichment of pathways related to GPCR signaling, behavior, hormone regulation, nitrogen compound metabolism, and protein secretion. (**C**) Volcano plot of DEGs comparing sham vs. HI at D1, showing minimal differential expression at this early injury stage. (**D**) GSEA identified a robust pathway enrichment at D1 post-HI, including *PI3K-Akt* signaling, *TGF-β*–related and profibrotic responses, metabolic rewiring, pluripotency, focal adhesion, spinal cord injury signatures, cell migration, immune activation, and regulation of neurogenesis. (**E**) Volcano plot comparing DEG in sham vs. HI mice at D5, revealing pronounced induction of ECM and fibrosis-associated genes (*Cd44*, *Bgn*, *Col11a1*, *Cnmd*, *Anxa5*). (**F**) GSEA and ORA of D5 HI vs. sham microglia, highlighting enrichment of *Wnt/FZD4* signaling, *PI3K-Akt–mTOR* pathways, and biological processes associated with fibrosis and ECM remodeling.

Transcriptomic differences observed between P10 (D1) and P15 (D5) mice were interpreted as normal developmental changes occurring over this period, since these animals did not undergo HI. In DEG analysis, neonatal microglia at D5 displayed the upregulation of *EGR1* (log_2_FC = 1.98, *p* = 0.01) and *ARG1* (log_2_FC = 5.87, *p* = 0.03) compared with D1 (Figure 3A), consistent with findings reported in previous studies [13,14]. GSEA indicated the activation of secretion and trafficking pathways, including protein secretion (GO:0009306, NES = 1.73, q = 0.065, *Cplx3*, *Sncg*, *EGR1*, *Rac1*) and cellular localization (GO:0051641, NES = 1.78, q = 0.19, *Slc17a7/Cplx3/Sncg/Scn4b/Rac1/Sox9/Nr4a3/Nectin3/Mertk/Zdhhc22*) (Figure 3B). Additional enriched processes included response to nitrogen compound (GO:1901698, NES = 1.77, q = 0.14, *EGR1/Rac1/Htr3a/Sox9/Nr4a3*) and regulation of hormone levels (GO:0010817, NES = 1.76, q = 0.14, *EGR1/Cplx3/Rac1*). The behavior-associated pathway (GO:0007610, NES = 1.79, q = 0.20) reflected the expression of *Slc17a7*, *Cux2*, *EGR1*, *Sncg*, and *Nr4a3*. Among WikiPathways, the GPCR Class A rhodopsin-like pathway showed negative enrichment (WP189, NES = −1.59, q = 0.09, *Opn3/Htr7/Oprl1/Ccr1*). ORA analysis identified the spinal cord injury pathway as the top term (WP2432, fold enrichment (FE)= 11.6, *p* = 0.006, *EGR1*, *ARG1*) (Figure 3B).

### 3.4. Early Transcriptomic Changes in Microglia on D1 Post-HI

No genes were identified by DEG analysis (Figure 3C). GSEA on D1 identified multiple significantly enriched biological processes (Figure 3D). The most enriched term was behavior (GO:0007610, NES = 1.97, q = 0.045, *Reln/EGR1/Cck/Slc17a7/Cux2/Sncg/Npy*), followed closely by signaling (GO:0023052, NES = 1.81, q = 0.047, *Pvalb/Cd44/Reln/EGR1/Cck/Slc17a7/Sncg/TGF-β1/Plce1/Npy/Rac1*). Pathways related to transcriptional regulation and metabolic activation were also significantly enriched, including regulation of gene expression pathway (GO:0010468, NES = 1.57, *p* = 0.008, q = 0.15, *Cd44/Reln/EGR1/Fos/Cux2/TGF-β1/Rorb/Sp7/Tshz2/Satb2/Prox1/Bmp3/Fli1*). Additionally, metabolic process regulation pathways such as positive regulation of cellular metabolic process (GO:0031325, NES = 1.68, q = 0.14, *Cd44/Reln/EGR1/Fos/Cux2/TGF-β1/Rorb/Rac1/Sp7/Satb2/Prox1/Bmp3/Fli1*) were enriched. Additional enrichment was observed for pathways associated with microglial morphology and motility, including regulation of cell projection organization (GO:0031344, NES = 1.83, q = 0.11; *Cd44*, *Reln*, *Cux2*, *Plce1*, *Rac1*) and cell migration (GO:0016477, NES = 1.78, q = 0.125; *Cd44*, *Reln*, *Cck*, *TGF-β1*, *Rac1*, *Satb2*, *Prox1*). Moderate enrichment was detected in immune-related pathways, including positive regulation of immune system process (GO:0002684, NES = 1.60, q = 0.16, *Cd44/Fos/TGF-β1/Rac1*). Broader developmental programs were represented by the tissue development pathway (GO:0009888, NES = 1.45, q = 0.20, *Cd44/Col1a2/EGR1/Fos/Bgn/TGF-β1/Rac1/Sp7/Satb2/Prox1/Cnmd/Pls1/Col11a1/Stat6*). Neurogenic and neuronal functional pathways were also significantly enriched, including positive regulation of neurogenesis (GO:0050769, NES = 1.62, q = 0.14, *Reln/Cux2/TGF-β1*) and gene sets linked to higher order neural functions such as learning or memory (GO:0007611, NES = 1.59, q = 0.16, *Reln/Slc17a7/Cux2*). GSEA using WikiPathways showed among the top enriched processes were spinal cord injury (NES = 1.65, q = 0.14; *EGR1*, *Fos*, *TGF-β1*), focal adhesion (NES = 1.63, q = 0.14; *Reln*, *Col1a2*, *Rac1*, *Col11a1*, *Lamc2*, *Tnr*, *Ccnd1*), and focal adhesion–PI3K–Akt–mTOR signaling (NES = 1.50, q = 0.14; *Reln*, *Col1a2*, *Col11a1*, *Csf3r*, *Lamc2*). Pathways related to pluripotency (NES = 1.50, q = 0.14; *Cd44*, *Fos*, *TGF-β1*, *Satb2*) and MAPK signaling (NES = 1.37, q = 0.23; *Fos*, *TGF-β1*, *Rac1*) were also enriched, consistent with the activation of regenerative and stress-response transcriptional programs (Figure 3D). GSEA of curated PMC pathways revealed that most significantly enriched signatures included cancer-promoting gene networks (NES = 1.84, q = 0.0008; *Cd44*, *Cck*, *Fos*, *Sncg*) and metabolic rewiring of the hypertensive kidney (NES = 1.72, q = 0.0046; *Cck*, *Fos*, *TGF-β1*, *Npy*). Further enrichment pathways included protein–protein interaction networks for the AGE–RAGE pathway (NES = 1.69, q = 0.044; *Col1a2*, *EGR1*, *TGF-β1*, *Plce1*, *Rac1*) and pro-fibrotic responses in cirrhosis (NES = 1.61, q = 0.054; *Cd44*, *Reln*). Multiple enriched datasets highlighted shared components of *TGF-β* and *Wnt* pathway regulation: *Wnt/FZD4* signaling via the miR-204 pathway (NES = 1.51, q = 0.12; *Cd44*, *TGF-β1*, *Rac1*) and concurrent *BMP* maintenance and *TGF-β* inhibition in resistance to muscle atrophy (NES = 1.45, q = 0.12; *Col1a2*, *TGF-β1*), enrichment of *PI3K–Akt* signaling–associated datasets (NES = 1.26; *Anxa5/TGF-β1*, *EGR1/Fos*). Key genes driving these patterns included *Pvalb* (log_2_FC 5.75, highly significant), *Cd44*, *Reln*, *Col1a2*, *EGR1*, *Cck*, *Fos*, and *Slc17a7* (Figure 3D).

### 3.5. Microglial Signatures on D5 Microglia Post-HI

DEG analysis identified a robust set of genes with significant changes in expression (Figure 3E). Among the most significantly upregulated genes in the HI group compared with SHAM controls were *Cd44* (log_2_FC = 3.23, adjusted *p* = 8.0 × 10^−4^), *Adssl1* (log_2_FC = 2.89, *p* = 0.0016), *Bgn* (log_2_FC = 2.92, *p* = 0.0038), and *Npy* (log_2_FC = 4.11, *p* = 0.0048). Additionally, several other genes such as *Col11a1* (log_2_FC = 2.56, *p* = 0.0093), *Anxa5* (log_2_FC = 2.15, *p* = 0.0226), and *AB124611* (log_2_FC = 3.085218463, *p* = 2.14 × 10^−4^) were upregulated. Conversely, *Lct* exhibited a significant downregulation (log_2_FC = −2.13, *p* = 0.0104). Other genes such as *Myo1f*, *Cnmd* showed moderate but significant expression changes (*p* < 0.05). GSEA of D5 microglia post-HI identified the top enriched gene set, annotated as aortic valve stenosis-related biological processes (NES = 1.72; q = 0.03, *Bgn*, *Col11a1*, *Fxyd1*, *TGF-β1*, *Plce1*). Several additional gene sets were also enriched, such as *PI3K/AKT*-related signatures (NES = 1.50, q = 0.11, *Anxa5/TGF-β1*) and *Wnt/FZD4*-linked signatures (NES = 1.44, q = 0.11, *Cd44/TGF-β1*) (Figure 3F). The ORA top enriched term was evidence of active pro-fibrotic response in blood of patients with cirrhosis (PMC4552880, FE = 9.22; z = 3.96; q = 0.097), driven by *Cd44* and *Col11a1.* A related ECM-associated term, aortic valve stenosis-related biological processes, also showed notable enrichment (PMC4934886, FE = 4.61; z = 2.51; q = 0.193), highlighting *Bgn* and *Col11a1* as overlapping components. Secondary, lower confidence enrichments were observed in pathways involving *PI3K/AKT* signaling (celastrol alleviates autoimmune hepatitis through the *PI3K/AKT* signaling pathway, FE= 5.53; q = 0.208, driven by *Anxa5*) and *Wnt/FZD4* signaling (FE= 4.61; q = 0.208, driven by *Cd44*). Finally, pathway-level mapping using the WikiPathways database revealed modest enrichment of focal adhesion (WP85; FE= 3.0; q = 0.358) and focal adhesion–*PI3K–AKT–mTOR* signaling (WP2841; FE = 2.45; q = 0.358), both involving *Col11a1.* Among the most strongly upregulated transcripts were *Cd44* (log_2_FC = 3.23, *p* = 4.1 × 10^−6^), *Bgn* (log_2_FC = 2.92, *p* = 5.9 × 10^−5^), and *Col11a1* (log_2_FC = 2.56, *p* = 2.8 × 10^−4^). In parallel, *TGF-β1* (log_2_FC = 1.55, *p* = 0.0057) and *Anxa5* (log_2_FC = 2.15, *p* = 9.2 × 10^−4^) were also upregulated. Additional upregulated genes were *Stat6* (log_2_FC = 1.52, *p* = 0.02), *Irf8* (log_2_FC = 1.42, *p* = 0.01), *Csf3r* (log_2_FC = 1.37, *p* = 0.02), *Npy* (log_2_FC = 4.11, *p* = 0.02), and *Vip* (log_2_FC = 2.04, *p* = 0.02).

**Figure 4:**
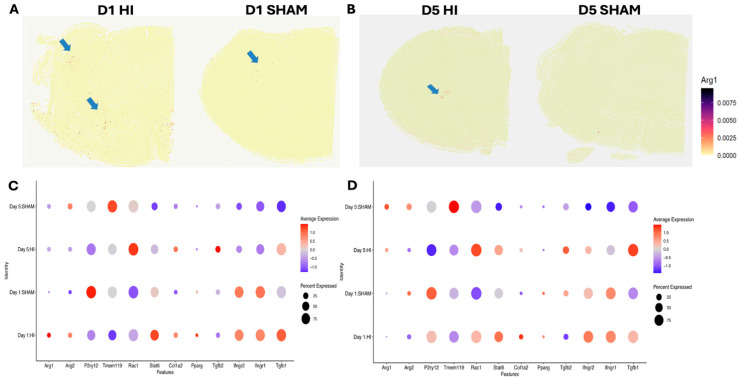
Expression patterns of selected genes post-HI. (**A**) Diffuse ARG1 expression in HI vs. SHAM on D1. (**B**) D5 ARG1 expression localizes to the HI hippocampus, while diminished during normal development. Expression of genes of interest in hemispheric microglia (**C**) and hippocampal microglia (**D**) across four conditions: D1 HI, D1 Sham, D5 HI, and D5 Sham. Color scale indicates average gene expression; bubble size represents the percentage of cells expressing each gene.

Microglia transition from an early inflammatory state at D1 to a matrix-remodeling revealed a *TGF-β* dominant phenotype with pro-fibrotic signaling at D5 post-HI. Analysis of hippocampal microglia across early and later post-injury timepoints revealed distinct spatial and temporal patterns of select genes associated with efferocytosis and fibrosis. Spatially, *ARG1* expression on D1 post-HI was diffusely distributed across the cortex, striatum, amygdala, and hippocampus (Figure 4A), whereas in sham mice it was restricted primarily to the globus pallidus and amygdala. On D5, *ARG1+* cells in HI animals localized predominantly to the hippocampus, while sham mice showed minimal to no *ARG1* expression (Figure 4B). In hemispheric microglia, both *ARG1* and *ARG2* were most prominently expressed on D1 post-HI, with *ARG2* increased on D5 in hemispheric microglia and on D1 and D5 in hippocampal microglia in sham animals (Figure 4C). In contrast, hippocampal microglia expressed very low levels of either ARG isoform on D1 post-HI, followed by a modest increase in *ARG1* on D5 post-HI and developmental upregulation of both isoforms in sham mice on D5. Microglial marker *P2RY12* was elevated on D1 in sham animals but declined with development and was markedly downregulated post-HI at both D1 and D5. *TMEM119* similarly increased developmentally in sham microglia at D5 and was suppressed by HI on D1. Genes involved in efferocytosis exhibited stage-specific activation. *STAT6* was induced on D1, whereas *Rac1* was elevated on D5; both were suppressed in sham animals. Fibrosis-related signaling also demonstrated temporal structuring. *TGF-β1* was strongly upregulated in hemispheric microglia on D1 post-HI, with a smaller increase in hippocampal microglia; by D5, *TGF-β1* decreased in the hemisphere but continued to rise in the hippocampus. *TGF-β2* was upregulated on D5 post-HI. *PPARγ* and *Col1a2* were increased in HI microglia on D1, declined by D5, but remained elevated relative to sham. HI microglia also expressed higher levels of *TGF-β2* on D5.

## 4. Discussion

This study defines the temporal and transcriptional landscape of neonatal microglial maturation and activation following brain HI. Through the integration of DEG, GSEA, ORA, and interactive enrichment analyses, we demonstrated that the neonatal microglial transcriptome undergoes a developmental transition to an *EGR1/ARG1*-driven state, while HI triggers an ECM-remodeling phenotype already at D1 post-HI.

Under physiological (sham) conditions, the transcriptional maturation of microglia from postnatal day 10 (D1) to day 15 (D5) was marked by a strong upregulation of *EGR1* and *ARG1*, consistent with their known roles in shaping neonatal neural circuits. *EGR1* is involved in processes essential to synaptic activity and plasticity including endocytosis, membrane organization, vesicle-mediated transport [33,34], and synaptic pruning [35]. *ARG1*-expressing microglia are known to contribute to the maturation of the cholinergic system and hippocampal synaptic plasticity [36]. *EGR1/ARG1* were also genes driving the strong enrichment of the spinal cord injury pathway in ORA analysis. This finding suggests that genes and regulatory networks pivotal for normal developmental functions can become reactivated during microglial response to injury, as seen in other studies [37].

Microglia rapidly deviated from this developmental trajectory post-HI. Within 24 h, they activated an injury-responsive transcriptional program enriched for efferocytosis, ECM remodeling, growth factor signaling, and metabolic adaptation, involving signaling cascades such as *PI3K–Akt–mTOR* pathway, *MAPK* [38], focal adhesion, and cell migration pathway. Consistent with this, we observed the increased expression of core efferocytosis and cytoskeletal remodeling genes (e.g., *Cd44*, *Rac1*, *TGF-β1*, *Anxa5*), which are all essential for efficient clearance of apoptotic cells [39,40,41], thereby reducing inflammation post-HI. *TGFβ1* has been shown to alleviate neuroinflammation through *PI3K/Akt* or *mTOR* signaling pathways [42]. In parallel, the enrichment of pro-fibrotic signatures (including “AGE–RAGE signaling” driven by *Cd44*, *Pvalb*, *Col1a2*, and *TGFβ1* genes) suggests that neonatal microglia rapidly adopt a phenotype bridging inflammation and tissue remodeling [43]. Notably, RAGE signaling has been identified in post-stroke ischemic brain associated with increased inflammation [44], supporting the relevance of this pathway in neonatal HI. This is consistent with prior evidence that early microglia can transiently adopt mesenchymal-like properties [45] and engage pro-fibrotic regulators such as *TGFβ* and *Wnt/FZD4*-related signaling pathways [46], positioning them at the interface of repair and scar formation after neonatal HI.

By D5 post-HI, this fibrotic program became more pronounced. *TGFβ2* and *Col11a1* were upregulated and an elevated expression of *Cd44* and *Bgn* indicated persistent ECM remodeling and structural repair [47]. *Col11a1* plays a role in ECM formation, particularly in collagen I, II, and XI synthesis [47]. GSEA and ORA analyses confirmed the activation of *Wnt/FZD4* signaling, *PI3K-Akt* signaling, and focal adhesion pathways on D1 and their persistent activation from D1 to D5, indicating a sustained commitment to cytoskeletal remodeling, motility, and tissue repair.

These three pathways intersect with the arginase axis, positioning *ARG1* as a central effector linking metabolic reprogramming with actin dynamics. Through L-arginine diversion and nitric oxide restriction, *ARG1* shapes FAK–Rho GTPase-dependent cytoskeletal organization [48], while *PI3K–Akt* signaling promotes *ARG1* expression to bias microglia toward a reparative phenotype and limit neurotoxic inflammation [49]. In contrast, *β-catenin* downstream of canonical *Wnt* signaling negatively regulates *ARG1* [50,51] and, depending on context, can either suppress microglial activation or potentiate proinflammatory responses [52]. Together, these findings suggest that microglia deploy a tightly regulated, multi-pathway network that balances *ARG1*-driven repair mechanisms with *Wnt-*mediated checks on activation, enabling a staged transition between acute injury responses and subacute remodeling.

Spatial mapping of *ARG1* revealed early, diffuse induction of *ARG1* across multiple brain regions on D1 consistent with widespread microglial activation post-HI. By D5, *ARG1* expression became regionally restricted to hippocampal and striatal zones that represent regions of delayed neuronal death [53]. In contrast, *ARG2* expression persisted under HI conditions, suggesting a distinct metabolic role for this isoform in injury resolution and ongoing oxidative stress. However, further work is necessary to fully elucidate the functional role of ARG2 in this context. While ARG1 is well-characterized for its anti-inflammatory functions in microglia [54], the role of ARG2 is less known. In the adult brain, ARG2 is required for shifting mitochondrial dynamics and bioenergetics towards an oxidative phenotype in inflammatory macrophages and, as a downstream mediator of IL-10, contributes to the resolution of inflammation [55].

Clinically, neonatal HI encephalopathy is characterized by evolving injury beyond the initial insult. The hippocampus is a key center for memory and learning deficits, and our spatial transcriptomic data suggest that microglial programs in this region evolve from early apoptotic clearance to subacute remodeling and fibrosis over days. This argues for time-sensitive and targeted therapeutic modulation. Interventions that enhance efficient efferocytosis and limit collateral inflammation during the acute period could be beneficial, and strategies that dampen excessive *TGFβ*-linked ECM deposition during the subacute period may reduce maladaptive fibrosis. Since these pathways also intersect with normal postnatal brain maturation, translational efforts will need to balance the promotion of repair with the preservation of developmental microglial functions.

Our study has several limitations. The sample size limits statistical power and the detection of subtle transcriptional changes, although integration analyses indicated minimal batch effects. Although microglial activation is expected early after HI, no individual DEGs were detected on D1, likely due to the limitation of the 220-gene targeted seqFISH panel. Pathway analyses nonetheless identified significant enrichments, supporting an early response consistent with the prior literature. The targeted seqFISH panel limits inference of M1 vs. M2 microglial polarization, highlighting the importance of employing expanded gene panels in future studies. While the Vannucci model is a well-established paradigm for neonatal HI, it does not fully capture the heterogeneous etiologies, systemic factors, or chronic injury evolution seen in human neonatal HI, and species-specific developmental differences may influence microglial and astrocyte responses. The seqFISH spatial transcriptomics platform is restricted by its targeted gene panel, preventing the detection of low-abundance transcripts or broader injury-induced pathways. Additionally, sex was not included as a biological variable. Pseudobulk differential expression analysis, while robust for sparse spatial datasets, may obscure cell-to-cell heterogeneity. Pathway enrichment tools also rely on curated databases that may include adult or non-neural annotations, complicating interpretation of some signatures.

An important conceptual limitation is that our model implicitly interprets the observed transcriptional programs as “transitions” of resident microglia, whereas accumulating evidence indicates a more complex interplay between microglia and infiltrating myeloid populations after CNS injury. In adult stroke, for example, *ARG1^+^* myeloid cells are preferentially confined to non-microglial, non-border-associated macrophage *CCR2*-lineage monocytes and localize with *Col1*-expressing fibroblasts in the peri-infarct scar, suggesting that perivascular fibroblasts may co-regulate myeloid recruitment and activation in development and disease [56,57,58]. Similar compartmentalization could occur after neonatal HI, but our current approach cannot definitively distinguish microglial from infiltrating myeloid states across all time points and regions. Finally, only two early time points (D1 and D5) were examined, limiting insight into the later stages of glial scarring, ECM remodeling, and long-term microglial states. Future studies incorporating larger cohorts, expanded gene panels, additional brain regions, and later time points will be required for a more comprehensive understanding of neonatal HI.

## 5. Conclusions

Extending the prior descriptions of a multi-phase HI response, our data resolve these phases at the microglial transcriptional level: an early efferocytosis program (*EGR1/ARG1/STAT6*) and a later matrix-remodeling program (*TGF1/2*, *Cd44*, *Col11a1*), each defining distinct therapeutic windows. This trajectory integrates developmental plasticity with injury-induced fibrotic programming, positioning microglia as central regulators of both neuroprotection and scarring. Fibrotic scarring that emerges along this trajectory plays a necessary short-term protective role but can become maladaptive if excessive or persistent. Shortly after HI, the deposition of collagen-rich ECM and the recruitment of perivascular fibroblasts help contain necrotic tissue, re-establish vascular integrity, and limit the spread of inflammatory cells and toxic metabolites into adjacent, still-viable parenchyma. At the same time, an overly dense or long-lasting fibrotic scar can impede axonal regeneration, restrict synaptic re-integration, and maintain a pro-inflammatory milieu that disrupts normal circuit maturation in the developing brain. In the neonatal context, where plasticity and ongoing developmental patterning are critical, microglia and other brain-infiltrating myeloid cells are positioned as central regulators of both neuroprotection and scarring. Modulating early pro-fibrotic signaling, particularly through the *TGF-β* and *PI3K/AKT* axes, may therefore offer therapeutic leverage to preserve the stabilizing aspects of fibroblast-driven scarring while limiting its chronic consolidation and the development of maladaptive fibrosis.

## Data Availability

All data generated or analyzed during this study are included in this article. Further enquiries can be directed to the corresponding author.

## Supplementary Materials

The following supporting information can be downloaded at: https://www.mdpi.com/article/10.3390/cells15030253/s1, Figure S1: Spatial transcriptomic quality control and dataset integration of HI and sham mouse brain samples at D1 and D5; Table S1: Total number of cells analyzed per sample; Table S2: Differentially expressed genes across developmental (D1 vs. D5 SHAM) and injury conditions (HI vs. SHAM at D1 and D5). Refs. [59,60,61,62,63,64,65,66,67,68] are cited in Supplementary Materials.
