# Supplementary material for: Temporal and Spatial Gene Expression Dynamics in Neonatal HI Hippocampus with Focus on Arginase
Source: Cells. 2026 Jan 28;15(3):253. doi: 10.3390/cells15030253 (PMC12896907; doi:10.3390/cells15030253)
Supplement: Supplementary file 1 [file cells-15-00253-s001.zip › Supplementary Material R- cells.pdf]

SUPPLEMENTARY MATERIAL

ANIMAL SUBJECTS

Strains used

We used wild-type C57BL/6 mice of both sexes. The animals were housed in the vivarium located in the Sandler Neurosciences Building at the University of California San Francisco. The UCSF Department of Animal Resources oversaw the care and maintenance of all animals.

Species: mice
Strain: C57BL/6
Age: P10–15
Sex: male and female

Procedures

Vannucci model of hypoxia–ischemia: All mice underwent hypoxic–ischemic injury under inhalational anesthesia at postnatal day 10 (P10). In mice, P9–P10 is considered a better rodent equivalent of the term human newborn. We applied our validated Vannucci model of brain HI, consisting of permanent coagulation of the left common carotid artery via a vertical incision under 4% isoflurane anesthesia to induce ischemia, followed by a 1- hour recovery period with the dam and subsequent exposure to 10% oxygen/balance nitrogen at 37 °C for 50 min to induce hypoxic–ischemic injury. The Vannucci model has been adjusted in other studies, with different oxygen concentrations and hypoxia durations used depending on the desired injury severity. For severe injury we routinely performed hypoxia in 10% oxygen/balanced nitrogen for 50 min. During surgical procedures, animals were placed on a heating pad to maintain body temperature. Once recovered, animals were returned to their home cage with their parents. After recovery from anesthesia, animals were closely monitored for body weight, activity, feeding, and maternal care. Any animal that appeared in distress (continuous weight loss, failure to grow, lethargy, excessive vocalization, freezing, lack of eating/drinking) was euthanized. We sought consultation with veterinary staff for any animal that exhibited >15% weight loss. For the experiments, animals were sacrificed at the study time points (P10 and P15), as required to study neurodevelopmental injury. We used the minimum number of animals necessary.

Minimization of pain and distress

Upon arrival to our facility, animals were checked and counted. Cages were tagged as “new animals” so that extra attention was paid to adaptation to the new environment. After surgery, cages were labeled with the date of surgery, and animals underwent additional health checks to confirm uneventful recovery and ensure timely pain treatment if needed. Veterinary care at our facility was provided at multiple levels. The Laboratory Animal Resource Center (LARC) veterinarians provided clinical services and oversight for UCSF research animals and ensured the humane use and care of all animals. For pain management and anesthesia, we followed LARC guidelines for UCSF laboratory animals, which conform to the recommendations of the American College of Laboratory Animal Medicine and the National Academies of Sciences. Discomfort and pain were limited to what was unavoidable. Isoflurane inhalation anesthesia delivered via a precision vaporizer allowed minute- by- minute titration during surgical procedures. Anesthesia was induced with 5–6% isoflurane and maintained with 1–3%

isoflurane. Anesthesia was combined with local analgesia using 0.25% bupivacaine applied topically to the incision site before incision and after surgery. After surgery the incision was disinfected, bupivacaine was reapplied, and the wound was closed using tissue glue. Wounds were monitored for dehiscence; if dehiscence occurred, the animal was re- anesthetized and the incision was sutured.

For euthanasia, we followed the policy issued by the UCSF Office of Ethics and Compliance–Institutional Animal Care and Use Program to ensure compliance with the Animal Welfare Act and Regulations, the Guide for the Care and Use of Laboratory Animals (8th Edition), and the AVMA Guidelines on Euthanasia. Only trained personnel performed euthanasia, and training was provided via individual or group workshops through the IACUC Training and Compliance program.

For current study, we used intraperitoneal injection of Euthasol as a terminal procedure. An intraperitoneal dose of at least 200 mg/kg sodium pentobarbital was administered, followed by either bilateral thoracotomy and perfusion with fixative or decapitation. We selected these methods because discomfort was limited to the initial anesthetic injection. Vascular perfusion was necessary for morphologic studies requiring optimal preservation of brain tissue. These methods were consistent with the recommendations of the American Veterinary Medical Association Panel on Euthanasia.

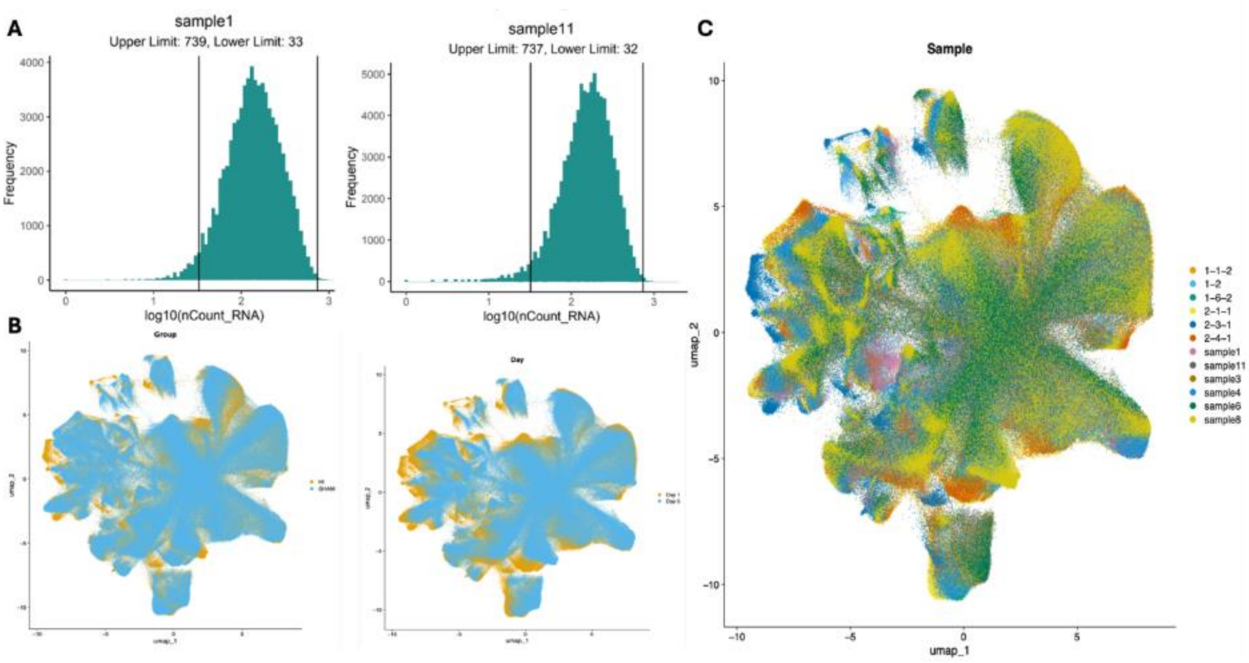

Figure S1: Spatial transcriptomic quality control and dataset integration of HI and sham mouse brain samples at D1 and D5.
(A) Histograms of total RNA counts per spot (log10-scaled) for two representative samples illustrate the upper and lower thresholds used for spot-level quality filtering. **(B)** UMAPs of all spots colored by Group (HI vs Sham) and Time (D1 vs D5) demonstrate successful integration and substantial overlap among conditions. **(C)** Combined UMAP of all samples colored by sample ID shows uniform distribution and minimal batch effect.

Sample ID	HI vs SHAM	Total number of cells analyzed

1-1-2	SHAM	66905
1-2-1	SHAM	64684
1-2-2	SHAM	63178
1-6-2	HI	83382
2-1-1	SHAM	73728
2-3-1	HI	54057
2-4-1	HI	71430
1	HI	73646
3	HI	63178
4	SHAM	85595
6	SHAM	87788
8	HI	90552
11	SHAM	90432

Table S1: Total number of cells analyzed per sample

D1 vs D5 SHAM			
**gene**	**log2FoldChange**	**pvalue**	**rank**

Egr1	1.981	5.52E-05	4.258
Arg1	5.874	2.86E-04	3.544
Lct	1.668	0.006	2.238
Slc17a7	1.83	0.007	2.183
Cplx3	2.814	0.008	2.081
Sncg	6.57	0.016	1.805
Tshz2	1.51	0.024	1.62
Arhgap29	1.351	0.025	1.609
Acot11	1.535	0.026	1.587
Cux2	1.318	0.028	1.55
Glce	1.184	0.032	1.501
Slc32a1	1.788	0.038	1.426
Scn4b	1.35	0.045	1.344
Rac1	1.209	0.047	1.33
Htr3a	1.984	0.049	1.313
Anxa5	1.265	0.053	1.279
Tmem119	1.527	0.054	1.271
**D1 HI vs SHAM**			
Pvalb	5.752	4.68E-04	3.33
Cd44	2.284	0.003	2.59
Reln	1.665	0.012	1.92
Col1a2	1.579	0.023	1.631
Egr1	1.15	0.037	1.427
Cck	2.011	0.04	1.4
Fos	1.304	0.053	1.277
Slc17a7	1.457	0.053	1.275
**D5 HI vs SHAM**			
Cd44	3.228	4.08E-06	5.389
Adssl1	2.893	1.61E-05	4.793
Bgn	2.916	5.86E-05	4.232
Npy	4.112	9.80E-05	4.009
AB124611	3.085	2.14E-04	3.669
Col11a1	2.562	2.84E-04	3.547
Anxa5	2.148	1.00E-03	3.035
Myo1f	1.739	0.001	2.947
Cnmd	2.526	0.002	2.73
Psmb8	1.946	0.005	2.27
Fxyd1	2.396	0.005	2.263
Tnr	1.722	0.006	2.241
Tgfb1	1.551	0.006	2.24
Plxdc1	1.518	0.008	2.096

Lag3	1.884	0.015	1.837
Irf8	1.424	0.017	1.778
Csf3r	1.375	0.019	1.728
Plce1	1.591	0.019	1.724
Stat6	1.511	0.02	1.697
Vip	2.043	0.023	1.633
Cplx3	2.04	0.024	1.614
Ccdc80	1.847	0.025	1.61
Pdpn	1.448	0.03	1.529
Sst	1.479	0.039	1.408
Serpinb8	1.344	0.049	1.309
Npnt	1.402	0.051	1.29

Table S2. Differentially expressed genes across developmental (D1 vs D5 SHAM) and injury conditions (HI vs SHAM at D1 and D5).
(1) developmental maturation in sham controls (D1 vs D5 SHAM), (2) early injury response (D1 HI vs SHAM), and (3) later injury response (D5 HI vs SHAM). For each gene, $\log_2$ fold change, p-value, and ranked significance are shown.

SUPPLEMENTARY DISCUSSION

Expanded details on different pathways and findings are provided here to maintain a focused main Discussion.

GSEA analysis of physiologic conditions in the shams further clarified microglial functions during this developmental window by identifying activation of pathways involved in protein secretion and cellular positioning. These pathway enrichments were driven in part by genes such as Cplx3 and Sncg [59, 60], which encode SNARE complex components essential for vesicle fusion, exocytosis, and intracellular trafficking. These mechanisms are consistent with microglia's role in shaping the synaptic and extracellular environment. We further observed that advancing maturation of the neonatal brain led to suppression of neuromodulatory GPCR pathways. During early development, GPCR pathways support axon guidance, cortical layer formation, and neuronal survival [61]; thus, we speculate that their downregulation suggests the transition from developmental plasticity toward more stable microglial and neuronal functions.

Consistent with other studies, the temporal regulation of efferocytosis-related genes highlighted an increase in STAT6 at the early timepoint after HI D1 [17], while Rac1 was elevated at later stages on D5 [62]. STAT6 upregulation at D1 likely reflects the early cytokine-driven response, where IL-4/IL-13–associated signaling promotes anti-inflammatory polarization [17], enhanced phagocytosis, and rapid transcriptional programs aimed at stabilizing the injured environment. By contrast, Rac1 elevation at D5 corresponds to the later remodeling phase, during which microglia engage with a progressively ECM-rich lesion core, requiring increased cytoskeletal dynamics, migration, and structural integration. Importantly, suppression of homeostatic microglial markers such as P2RY12 and TMEM119 highlights a loss of classical microglial identity following injury [63,64].

HI triggered a rapid immune response marked by a widespread surge of TGFβ1 in the hemisphere during the acute phase. At later stages, TGFβ expression became concentrated in the hippocampus, a region characterized by delayed neuronal vulnerability. TGFβ1 often acts synergistically with TGFβ2 to promote fibrosis [65], and the delayed rise in TGFβ2 suggests a shift toward its dominance during the subacute phase, potentially driving the chronic components of glial scar formation. The increased expression of PPARγ and Col1a2 reflects downstream activation of TGFβ signaling [66,67] and supports a microglial shift toward tissue remodeling after HI. Col1a2, a core structural component of Type I collagen, was strongly upregulated on D1, indicating rapid initiation of a pro-fibrotic ECM deposition program. Although its levels declined by D5, they remained elevated relative to sham, consistent with ongoing matrix remodeling and early scar formation. PPARγ showed a similar temporal pattern—peaking on D1 and decreasing by D5 while still exceeding sham levels. This early induction likely reflects the metabolic reprogramming required for microglial activation, motility, and phagocytosis, while its sustained expression suggests persistent engagement in inflammation modulation and lipid clearance within the evolving injury environment [68].
